# Genetic structure, antimicrobial resistance and frequency of human associated *Escherichia coli* sequence types among faecal isolates from healthy dogs and cats living in Canberra, Australia

**DOI:** 10.1371/journal.pone.0212867

**Published:** 2019-03-04

**Authors:** Judith A. Bourne, Wye Li Chong, David M. Gordon

**Affiliations:** 1 Ecology and Evolution, Research School of Biology, the Australian National University, Acton, Australian Capital Territory, Australia; 2 RSPCA Veterinary Clinic, Wright, Australian Capital Territory, Australia; The Pennsylvania State University, UNITED STATES

## Abstract

Extraintestinal pathogenic *Escherichia coli* (ExPEC) cause clinical infections in humans. Understanding the evolution and dissemination of ExPEC strains via potential reservoirs is important due to associated morbidity, health care costs and mortality. To further understanding this survey has examined isolates recovered from the faeces of 221 healthy dogs and 427 healthy cats. The distribution of phylogroups varied with host species, and depended on whether the animal was living in a shelter or a home. The human associated STs 69, 73, 95, 131 and 127 were prevalent, with 30.5% of cat isolates and 10.3% of dog isolates representing these ExPEC sequence types. Resistance to the antibiotics ampicillin and tetracycline was common, but resistance to other antimicrobials was negligible.

## Introduction

*Escherichia coli* is a common member of the microbiota of the lower intestine of mammals and to a lesser extent birds [1]. The species is genetically diverse and exhibits considerable genetic substructure [2]. The species has been partitioned into eight phylogroups [3]. *E*. *coli* isolates are most likely to belong to phylogroups A, B1, B2 and D, while strains belonging to phylogroup C, E, F and Clade I are less frequently observed [1]. Strains belonging to the various phylogroups differ in their genome size, variable gene content, disease association, ecological niche, and life history characteristics [4].

Although most strains of *E*. *coli* behave as commensals of the lower intestine of vertebrates, some are able to cause intestinal and extra-intestinal disease. Strains responsible for intestinal disease have diverse phylogenetic origins [5]. However, most strains responsible for extra-intestinal diseases such as urinary tract infections (UTIs) or septicaemia are members of phylogroup B2 and to a lesser extent phylogroup D [6, 7]. The technique of multi-locus sequence typing (MLST) [8] has revealed that there are hundreds, of sequence types (STs) within phylogroups B2 and D. However despite this diversity, a very small fraction of STs, are responsible for the great majority of extra-intestinal infections. These are phylogroup B2 STs, ST73, ST95, and ST131 and the phylogroup D ST, ST69, which are largely human associated [9]. At least one (ST 95) is rarely isolated from livestock, wild birds or mammals [10]. The frequency with which these common human associated STs are observed in companion animals is unclear.

Extra-intestinal infections also occur in dogs and cats, primarily as uncomplicated UTIs. As in humans, *E*. *coli* is the most common cause of such infections in dogs [11]. Urinary tract infection is relatively uncommon in cats [12], but when it does occur, *E*. *coli* is also the most common cause. As in humans, isolates belonging to phylogroup B2 and D are more likely to be recovered from extra-intestinal infections of companion animals [13, 14]. The four STs commonly responsible for extra-intestinal infection in humans (STs 69, 73, 95 and 131) have been isolated from healthy or diseased companion animals. ST131 is commonly reported in dogs and cats [15–17] but there are few reports of the other STs: ST69 in dogs, ST73 in cats and dogs, ST127 in dogs and a single ST95 isolate in a dog [18–21]. What studies have been undertaken have largely been based on clinical isolates exhibiting resistance to particular antimicrobials, such as fluoroquinolones [15, 17, 22].

Although the human associated STs 69, 73, 95 and 131 have been observed in companion animals, there is relatively little data with which to assess the extent to which companion animals represent a zoonotic reservoir of these human associated STs. Family members, including the family pet, have been shown to be more likely to share *E*. *coli* strains than individuals not living in the same household [23]. While, in one study, an ST73 strain was found to be shared by family members, including the dog, and persisted in the family for more than three years [24].

The aim of the present study was to assess the genetic structure, diversity, as well as the frequency of the human associated STs of *E*. *coli* in the faeces of dogs and cats living in the Canberra region of Australia, and to determine the antibiotic sensitivity of the isolates recovered.

## Results

### *E*. *coli* diversity

*E*. *coli* was detected in 334 (78.2%) of 427 cats and in 203 (91.9%) of 221 dogs. Thus *E*. *coli* was significantly less likely to be detected in cats as compared to dogs (Contingency Table Analysis: P>X^2^ < 0.001).

The relative abundance of the phylogroups varied with host type (cat or dog) and differed depending on if the animal was a pet or residing in an animal shelter (Nominal Logistic Regression: Cat/Dog, P>X^2^ < 0.01; Pet/Shelter P>X^2^ < 0.001: Cat/Dog*Pet/Shelter P>X^2^ < 0.82) (Table 1). There was little difference in the relative abundance of phylogroup B1, D, E or F strains in cats compared to dogs, but phylogroup B2 strains were overrepresented in cats, while phylogroup A were overrepresented in dogs (Table 1). Contrasting cats and dogs living in an animal shelter with those living in a home reveals that individuals living in a shelter have lower frequency of phylogroup A strains and an increased frequency of B1 strains compared to animals not living in a shelter (Table 1). The relative abundance of phylogroups B2, D, E and F did not differ between animals living in a home versus those residing in a shelter.

**Table 1:** The relative abundance of *E*. *coli* phylogroups among 203 *E*. *coli* isolates from 221 dogs and 334 isolates from 427 cats.

Phylogroup	Cat		Dog	
	Pet % (n)	Shelter % (n)	Pet % (n)	Shelter % (n)
A	15.6 (10)	5.6 (15)	31.8 (55)	13.3 (4)
B1	26.6 (17)	41.6 (112)	33.0 (57)	46.7 (14)
B2	40.6 (26)	41.6 (112)	24.3 (42)	33.3 (10)
D	6.2 (4)	7.4 (20)	6.4 (11)	6.7 (2)
E	0 (0)	0.3 (1)	1.2 (2)	0 (0)
F	10.9 (7)	3.7 (10)	3.5 (6)	0 (0)

(n), number of samples.

For cats residing in an animal shelter the relative abundance of the phylogroups did not vary with the sex of the animal (Contingency Table Analysis: P>X^2^ < 0.16)(data not presented). For dogs, the relative abundance of the phylogroups did not differ between the sexes (Nominal Logistic Regression: Sex, P>X^2^ < 0.051; Pet/Shelter P>X^2^ < 0.12) (data not presented).

Of the 334 isolates from cats, 30.5% represented one of the human associated STs (STs 69, 73, 95, 127, 131), a significantly greater frequency of human associated STs than was observed in dogs, as only 10.3% of the 203 isolates from dogs represented these STs (Contingency Table Analysis: P>X^2^ < 0.0001).

If either a cat or dog harboured a phylogroup D strain, then the likelihood that the strain was an ST69 strain did not depend on the host from which the isolate was recovered (Contingency Table Analysis; P>X^2^ = 0.17)(Table 2). Similarly, if either a cat or dog harboured a phylogroup B2 strain, then the likelihood that phylogroup B2 strain was an ST95, ST127 or ST131 strain did not depend on the host species from which the isolate was recovered (Contingency Table Analysis; ST95, P>X^2^ = 0.09; ST127, P>X^2^ = 0.19; ST131, P>X^2^ = 0.15)(Table 2). However, a phylogroup B2 strain was significantly more likely to be an ST73 strain if it was recovered from a cat rather than isolated from a dog (Contingency Table Analysis; P>X^2^ < 0.0001)(Table 2).

**Table 2:** Frequency of major human associated *E*. *coli* sequence types (STs) with respect to either the number of phylogroup D isolates (ST69), the number of B2 isolates (STs 73, 95, 127, 131) or the total number of *E*. *coli* isolates recovered from the host group.

Phylogroup	ST	Cat (n)	% of D or B2 isolates	% of *E*. *coli* isolates	Dog (n)	% of D or B2 isolates	% of *E*. *coli* isolates
D	69	8	33.3	2.4	2	15.4	1.0
B2	73	64	46.4	19.1	8	15.4	3.9
B2	95	24	17.4	6.9	4	7.7	2.0
B2	127	6	4.3	1.8	5	9.6	2.5
B2	131	1	0.7	0.3	2	3.8	1.0

ST, sequence type; n, number of samples.

Overall genotype (REP-type) diversity was high, indicating that most animals hosted a different genotype (Table 3). There were two exceptions to this pattern, phylogroup B2 and F isolates from cats. The lower levels of diversity observed among the B2 isolates was largely due to the high frequency of ST73 strains, while a single genotype represented 70% of phylogroup F isolates detected.

**Table 3:** *E*. *coli* genotype (REP = types) richness and diversity of *E*. *coli* isolated from cats and dogs with respect to the phylogroup membership of the isolate.

Phylogroup	Cat			Dog		
	Number of Isolates	Number of Rep Types	Simpson Diversity	Number of Isolates	Number of Rep Types	Simpson Diversity
A	25	22	0.99	59	38	0.98
B1	129	64	0.98	71	67	0.99
B2	138	71	0.94	52	35	0.97
D	24	21	0.99	13	12	0.99
F	17	6	0.51	6	6	1.00

### Antimicrobial sensitivity

Excepting ampicillin and tetracycline, most isolates were susceptible to all of the antibiotics tested (Table 4). Among cats and dogs with a home, 62.5% of 64 isolates from cats were susceptible to both ampicillin and tetracycline, while 70.5% of 173 dog isolates were susceptible and there was no difference in the ampicillin/tetracycline sensitivity profiles of isolates from cats or dogs with a home (Contingency Table Analysis: P>X^2^ = 0.30), and resistance to both ampicillin/tetracycline was uncommon (3.4%). Among the dogs and cats residing in an animal shelter 65.2% of 270 isolates from cats were sensitive to both ampicillin and tetracycline, while 83.3% of 30 dog isolates were susceptible. There was a significant difference in the ampicillin/tetracycline sensitivity profiles of isolates from animals residing in a shelter (Contingency Table Analysis: P>X^2^ = 0.034), with dog isolates more likely to be sensitive to both antibiotics, and while 10% of isolates from cats were resistant to both ampicillin and tetracycline, none of the dog isolates were resistant to both antibiotics.

**Table 4:** Antibiotic disc diffusion test results for 334 *E*. *coli* isolates from cats and 203 isolates from dogs.

Antibiotic	Cat % resistant (n)	Dog % resistant (n)
Ampicillin	31.1 (104)	20.2(41)
Tetracycline	13.2 (44)	9.9 (20)
Chloramphenicol	0.3 (1)	0
Ceftazidine	0.3 (1)	0.5 (1)
Nalidixic acid	0.3 (1)	0.5 (1)
Gentamicin	0	0.5 (1)
Ciprofloxacin	0	0.5 (1)
Ertapenem	0	0

(n), number of samples

**Fig 1:**
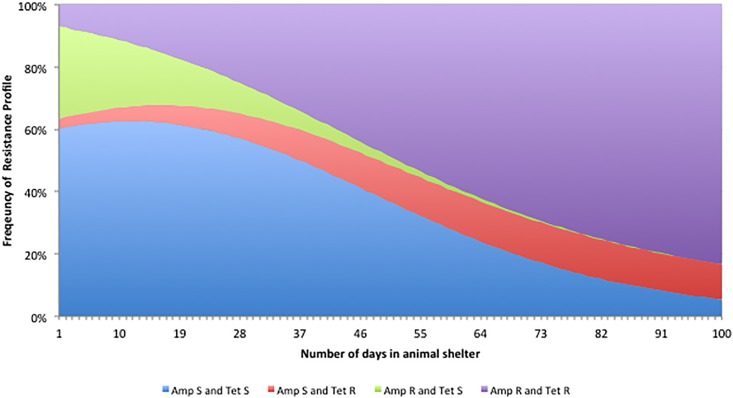
Predicted change in the frequency of *E*. *coli* isolates from cats resistant to ampicillin, tetracycline, or both antimicrobials as a function of the numbers of days the cat had resided in the animal shelter.

The frequency of isolates susceptible to both ampicillin and tetracycline declined significantly with the number of days the cat spent in the animal shelter, while the frequency of isolates resistant to both antibiotics increased (Nominal Logistic Regression: P>X^2^ = 0.004)(Fig 1). There was a modest increase in the frequency of isolates resistant to just tetracycline (Fig 1).

## Discussion

Few other studies have examined the relative abundance of the *E*. *coli* phylogroups in the faeces of healthy dogs and cats, and comparison of these studies reveals little concordance among studies (Table 5). The relative abundance of phylogroups in human faecal samples has been shown to vary with geographic locality [25]: in samples taken from humans living in temperate climates/developed countries, isolates belonging to phylogroup B2 are most common, while phylogroup A or B1 strains dominate in samples from people living in tropical climates/developing countries [25, 26]. All of the studies investigating the genetic structure of *E*. *coli* in healthy dogs were in temperate climate/developed country locations. While, typical dog food diets may vary between Japan and the other countries, it seems unlikely that there are large differences among the diets fed to dogs in Australia, Canada and the United Kingdom. The present study has shown differences in phylogroup structure depending on if the dogs were household pets or residing in an animal shelter. The dogs sampled in the Canadian and UK studies were household pets. In healthy cats living in Toronto, Canada, faecal *E*. *coli* phylogroup representation was 76.2%, 14.3%, 9.5% and 21% respectively for A, B1, B2 and D phylogroups [27]. Phylogroup representation of *E*. *coli* isolated from rectal swabs of healthy cats in Iran (n = 90) of groups A, B1, B2 and D was 66.7%, 1.2%, 13.4% and 18.9% [28]. By contrast, the frequency of phylogroup B1 and B2 strains in Australian cats was much higher (Table 1). Further studies are required if we are to understand the differences in phylogroup structure of *E*. *coli* populations isolated from a single host species sampled from different localities.

**Table 5:** Relative abundance of *E*. *coli* phylogroups isolated from healthy dogs living in different countries.

Phylogroup	Phylogroup (%)			
A	7.1	7.4	12.2	29.1
B1	53.6	25.0	58.8	35.0
B2	14.2	41.2	23.7	25.6
D^a^	21.4	26.4	5.3	10.3
Number of dogs	28	34	73	203
Locality	Canada	Japan	United Kingdom	Australia
Reference^b^	[27]	[29]	[30]	This study

^a^ Phylogroup E and F strains detected using the Clermont [31] method would be identified as D strains using Clermont [3].

^b^ Published studies used the Clermont (2000) [31] method

REP typing revealed a high diversity amongst dominant isolates. Overall there were 185 unique DNA fingerprints among 334 cat isolates, and 158 unique types among the 201 dog isolates. This result is comparable with that reported by [32] with 48 unique types among 108 isolates from 37 cats, and 106 types among 196 isolates from 71 dogs.

The human associated ExPEC STs represent about a third of all *E*. *coli* isolates recovered from human faecal samples in the Canberra region [[10]; unpublished data] (Table 6). Dogs are as likely as people to carry ST127 strains, but substantially less likely to carry strains belonging to the other human associated STs (Table 6). By contrast, while cats are less likely to harbour ST95, ST131 or ST69 strains, they are as likely to harbour ST127 strains and are almost twice as likely to carry ST73 strains compared to carriage of these STs in humans (Table 6).

**Table 6:** Frequency with which STs 69, 73, 95, 127, and 131 are detected in cats, dogs and people living the in Canberra, ACT region of Australia.

Phylogroup	ST	Human % of *E*. *coli* isolates	Cat % of *E*. *coli* isolates	Dog % of *E*. *coli* isolates
**D**	69	4.6	2.4	1.0
**B2**	73	10.0	19.1	3.9
**B2**	95	9.8	7.2	2.0
**B2**	127	2.0	1.8	2.5
**B2**	131	7.8	0.3	1.0

All of the human associated ExPEC STs have been isolated from healthy or diseased dogs, but the great majority of the previous studies focused on clinical isolates, used antibiotic selection (especially fluoroquinolones), or both. ST131 is the most common ExPEC ST reported in dogs and cats [15, 16, 33] but there are a few reports of the other STs: ST69 in dogs, ST73 in cats and dogs, ST127 in dogs and a single ST95 isolate in a dog [18–20].

There is concern that antimicrobial resistance in *E*. *coli* harboured by companion animal strains may transfer to humans further limiting treatment options for ExPEC infections in humans. While admitting that there is no direct evidence of transmission of antimicrobial resistance from *E*. *coli* in pets to humans, several authors [34–36] point out that the close relationship of humans and their companion animals provides opportunities for sharing strains; that resistant bacteria and genes have been recovered from healthy pets [35]; that the same classes of antimicrobials are used in human and veterinary medicine [37]; and cases of possible ExPEC transmission from pet animals to humans have been reported [38, 39].

Screening of dominant *E*. *coli* isolates in this study found phenotypic resistance to ampicillin in 31.1% cat isolates and 20.2% of dog isolates and to tetracycline in 13.2% cat isolates and 9.9% dog isolates. Resistance to ampicillin and tetracycline is common and this finding is consistent with most previous studies. A recent survey of *E*. *coli* faecal isolates from dogs visiting veterinarians in the UK revealed that 37.2% of isolates were resistant to ampicillin and 30% were resistant to tetracycline [40]. A survey of dogs and cats in Portugal found a much lower prevalence of ampicillin resistance in *E*. *coli* faecal isolates with 16.7% of cat isolates resistant and 7.7% of dog isolates resistant: levels of resistance to tetracycline were 20.5% for faecal isolates from dog isolates and 18.2% for cat faecal isolates [35]. A survey of healthy kennel dogs in Belgium found 12% resistance to ampicillin and 17% resistance to tetracycline and very low or not detectable levels of resistance to chloramphenicol, enrofloxacin and gentamicin [41]. A Canadian study of clinical *E*. *coli* isolates from canine UTI infections found low levels of resistance to all antibiotics, with ampicillin at 8.8% and tetracycline at 7.1% and no extended-spectrum beta-lactamases were detected [42]. Other recent studies of clinical *E*. *coli* isolates from UTI infections in dogs and cats reported resistance to a wider range of antibiotics, including quinolones and extended-spectrum beta-lactamases (ESBL) [43, 44].

Direct contact with companion animals living in close contact with their owners, has been implicated as a source of human infection with extended spectrum beta-lactamase (ESBLs) producing *E*. *coli* [23, 45]. However in this study very few *E*. *coli* isolates from dogs and cats were resistant to third generation cephalosporins, based on screening using ceftazidime. This antibiotic is considered one of the third generation cephalosporins with the highest sensitivity for ESBL detection [46]. The ESBL susceptibility found in this study contrasts with reports elsewhere in Australia and overseas of high levels of ESBL resistance in *E*. *coli* isolated from both dogs and cats [17, 40, 47–54]. However these studies used selective media or clinical isolates from diverse sites.

In common with another report which didn’t use antibiotic selective media [55], there was no evidence of resistance to carbapenems among isolates from Canberra dogs and cats.

The frequency of *E*. *coli* isolates from cats resistant to both ampicillin and tetracycline, increased with the time animals had resided in the animal shelter prior to being sampled. Tetracyclines (doxycycline) were commonly used to empirically treat cats residing in the shelter showing symptoms of feline upper respiratory infection (causal agents include feline herpesvirus, calicivirus, *Mycoplasma felis*, *Chlamydophila felis* and *Bordatella bonchiseptica*) and secondary bacterial infection as recommended by the Australian Infectious Diseases Advisory Panel—Antibiotics (http://www.ava.com.au/sites/default/files/AVA_website/pdfs/AIDAP%20prescribing%20guidelines.pdf). Isolates resistant to ampicillin, tetracycline or both were observed among strains belonging to phylogroups A, B1, B2, D and F. Such outcomes suggest that the evolution of isolates resistant to both antibiotics may have been due to the transfer of an R plasmid among isolates belonging to different lineages, rather than independent evolution events occurring in isolates belonging to each of the phylogroups. Sequential sampling of the same individuals through their stay at the animal shelter coupled with whole genome sequencing of the isolates would be required to determine how the frequency of isolates resistant to both antibiotics arises.

This study has shown that companion animals do carry *E*. *coli* isolates belonging to one of the human associated lineages. However considerable substructure is known to occur within clonal complexes 73, 95, and 131 [10, 56, 57]. Clonal complex 95 consists of at least five well-defined subgroups and while CC95 strains can be isolated from poultry and humans [10], the great majority of isolates from poultry belong to just one of the CC95 subgroups. Such an outcome suggests that there may be some degree of ‘host preference’ exhibited by isolates belonging to the same clonal complex. Substructure is also present in ST131 [57] and ST73 [56] but clear host specificities have not been described to date [58, 59]. Whole genome sequencing studies are required in order to determine the degree of difference exhibited by isolates belonging to the same clonal complex but isolated from different host species.

## Materials and methods

### Source of samples

Faecal samples from 221 dogs and 427 cats in the city of Canberra and its surrounds (Australian Capital Territory, Australia) were collected from 2015 to 2017. Cat samples were collected from an animal shelter and short-term boarding facilities. Dog samples were either collected by the owner, from domestic dogs at a dog park, at short-term boarding kennels or at longer-term kennels; including dogs housed at an animal shelter. Host age and body mass was not available for most animals, but the isolates were generally taken from adults.

### *E*. *coli* isolation

Fresh faecal material was dilution streaked onto MacConkey agar plates [60] and incubated overnight at 35°C. One well isolated lactose-positive colony from each sample was transferred to Simmons citrate and Luria Bertani agar plates [60] and incubated overnight at 35°C. Putative *E*. *coli* isolates (lactose positive and citrate negative) were confirmed to be *E*. *coli* by genetic analysis (see below). Isolates were inoculated into 5 ml of lysogeny broth (LB) and incubated at 36°C with shaking for 18 h. A 1 ml aliquot of this suspension was combined with 0.5 ml sterile glycerol and stored at -80°C.

### *E*. *coli* characterisation

The isolation procedure yielded 203 isolates from 221 dogs and 334 isolates from 427 cats. DNA was extracted from an overnight LB culture of each isolate using DNAzol genomic DNA isolation reagent (Invitrogen) according to the manufacturer’s instructions. Ethanol precipitated DNA was resuspended in Tris-EDTA buffer and this template DNA was stored at -20C.

DNA fingerprints of different strains were obtained using polymerase chain reaction (PCR) to amplify Repetitive Extragenic Palindromic (REP) elements and Enterobacterial Repetitive Intergenic Consensus (ERIC) sequences [61, 62]. PCR amplification was performed in an Applied Biosystems 2720 Thermal Cycler. REP PCR reaction mix (20 μl) contained about 10 ng of DNA template, 1 x PCR polymerisation buffer (Fisher Biotech), MgCl2 at 3.5 mM, N_6_(CGG)_4_ primer [62] at 0.4 mM and 1.0 U MyTaq Red DNA polymerase (Bioline). Cycling conditions were as follows: denaturation at 95°C for 3 min, followed by amplification for 35 cycles of 95°C for 1 min and 72°C for 3 min, followed by a final extension step at 72°C for 8 min. The ERIC PCR reaction mix (20 μl) contained approximately 10 ng of DNA template, 1 x PCR polymerisation buffer (Bioline), ERIC1 and ERIC2 [61] each at 0.4 mM and 1.0 U MyTaq Red DNA polymerase (Bioline). ERIC cycling conditions were as follows: 2 min initial denaturation at 95°C, followed by 30 cycles of 3 s at 94°C, 30 s at 92°C, annealing at 50°C for 1 min and extension at 65°C for 8 min. PCR products were separated and visualised on 1.2% agarose gels in 1-Trisborate-EDTA (TBE) buffer using ethidium bromide staining.

Isolates were assigned to a phylogroup using the method of Clermont [3]. The reaction mix (20 μl) contained about 10 ng of DNA template, 1 x MyTaq Red PCR buffer (Bioline), Primers (arpA.f, arpA.r, Chua.1b, Chua.2, Yja.1b, Yja.2b, Tsp.1b and Tsp.2 each at 0.4 mM and 1.0 U MyTaq Red DNA polymerase (Bioline). Cycling conditions were as follows: 5 min initial denaturation at 94°C, followed by 30 cycles of 5 s at 94°C, 25 s at 59°C; with a final extension of 5 min at 72°C. PCR products were separated and visualised on 1.2% agarose gels in 1-Trisborate-EDTA (TBE) buffer using ethidium bromide staining.

Phylogroup B2 and D isolates were screened for the presence of 4 human associated sequence types (STs) 69, 73, 95 and 131 using the method of Doumith [9]. The method of Clermont [63] was used to detect whether phylogroup B2 isolates could be assigned to any of the nine main *E*. *coli* phylogroup B2 lineages involved in human extra-intestinal infection strains.

### Antimicrobial susceptibility

All isolates were screened using the BD BBL Sensi-Disc procedure (http://legacy.bd.com/ds/technicalCenter/inserts/8840621(201107).pdf) on Mueller-Hinton agar plates (Merck) and using BBL Sensi-Disc antimicrobial susceptibility test discs (Becton, Dickinson and Company). The antimicrobials representing eight key antimicrobial classes were ampicillin (10 μg), ciprofloxacin (5 μg), tetracycline (30 μg), chloramphenicol (30 μg), gentamicin (120 μg), ertapenem (10 μg), nalidixic acid (30 μg) and ceftazidine (30 μg). The zone of inhibition diameters were measured using the instrument ProtoCol 3 (Synbiosis). The *E*. *coli* strains were classified as susceptible, intermediate or resistant to an antimicrobial, based on their zone diameters after 18 to 24 hours incubation at 35°C using BBL Sensi-Disc breakpoints.

Host metadata and REP type designation, phylogroup, human-associated ST, and antimicrobial susceptibility profile for all Escherichia coli isolates are presented in S1 Table.

## Supporting information

S1 Table Host metadata and REP type designation, phylogroup, human-associated ST, and antimicrobial susceptibility profile for all Escherichia coli isolates. (XLSX)
